# Facile synthesis of 1D organic–inorganic perovskite micro-belts with high water stability for sensing and photonic applications†
†Electronic supplementary information (ESI) available. CCDC 1862717. For ESI and crystallographic data in CIF or other electronic format see DOI: 10.1039/c9sc00162j


**DOI:** 10.1039/c9sc00162j

**Published:** 2019-03-08

**Authors:** Xiaogang Yang, Lu-Fang Ma, Dongpeng Yan

**Affiliations:** a Beijing Key Laboratory of Energy Conversion and Storage Materials , College of Chemistry , Beijing Normal University , Beijing 100875 , P. R. China . Email: yandp@bnu.edu.cn ; Fax: +86-10-6442-5385; b College of Chemistry and Chemical Engineering , Henan Province Function-oriented Porous Materials Key Laboratory , Luoyang Normal University , Luoyang 471934 , P. R. China

## Abstract

The development of low-dimensional perovskite micro/nanostructures with high water stability for novel photonic/electronic applications is highly desirable. Herein, one-dimensional (1D) organic–inorganic hybrid perovskite micro-belts [(AD)Pb_2_Cl_5_] (OIHP-AD, AD = acridine) were facilely synthesized through fast precipitation in aqueous solution at room temperature without any organic solvent and expensive alkyl halide. Luminescent properties and water stability are efficiently enhanced due to the highly regular arrangement of the protonated AD dyes with larger steric hindrance distributed in the perovskite host–guest system, which can afford denser crystal packing to prevent water erosion. The OIHP-AD micro-belts present upconversion fluorescence, polarized photoemission and optical waveguide performances with a low loss coefficient (0.004 dB μm^–1^) during propagation, thus extending the applications of 1D perovskite micro/nanostructures to potential optical communication micro-devices.

## Abstract 2

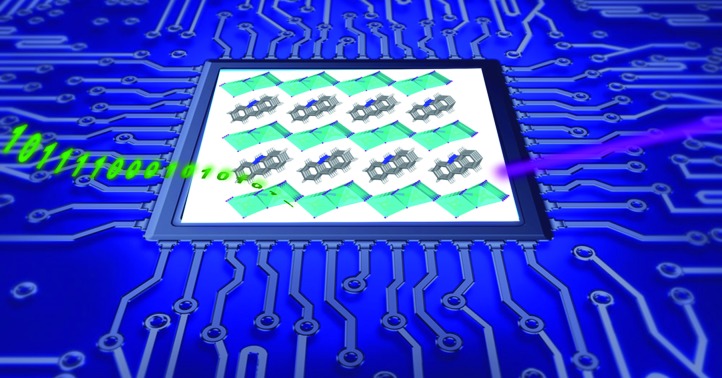
The development of low-dimensional perovskite micro/nanostructures with high water stability for novel photonic/electronic applications is highly desirable.

## Introduction

Rational design and controlled synthesis of perovskite materials have been extensively studied in recent years due to their tunable band gaps, superior charge-transfer properties, and potential applications in optoelectronic fields such as solar cells,^1^ photocatalysis,^2^ photodetectors,^3^ light-emitting diodes (LEDs),^4^ optically pumped lasers,^5^*etc.*^6^,^7^ Additionally, the high optical gain properties of these perovskite materials can extend their use as optical and photonic devices.^8^ To date, several challenges remain in the development of organic–inorganic hybrid perovskite systems. For example, their water stability is still relatively low, which has largely restricted their practical applications. On the other hand, due to their quantum-size effects compared with bulk materials, great attention has been focused on the fabrication of micro-/nano-scale perovskite crystals.^9^ However, both facile and large-scale synthesis of high-quality low-dimensional perovskites with novel photo-electronic performance is still relatively limited.

Generally, the optical properties of molecular materials are governed by pure chromophores (single molecule states) and their arrangement/packing fashion (aggregate states). In this context, the organic–inorganic hybrid perovskite structure is an idealized host–guest system for the formation of highly ordered chromophores and the uniform orientation of transition dipole moments for the construction of micro-/nano-scale optical devices. In such hybrid perovskite materials, organic cations play an important role in the light harvesting process. Herein, a rigid lead chloride inorganic matrix was selected as the typical 2D perovskite host layer; the acridine (AD) molecule was selected as the guest unit due to its excellent optical properties and wide applications for coloration and cell cycle determination.^10^ The AD molecule exhibits several polymorphic forms and has a tendency to be protonated in the presence of organic/inorganic acids.^11^ In addition, strong electrostatic and hydrogen bond interactions exist between the host–guest components. The larger steric hindrance of AD cations distributed between the rigid lead chloride inorganic matrixes can effectively prevent water erosion and maintain long-term stability under humid conditions.

**Scheme 1:**
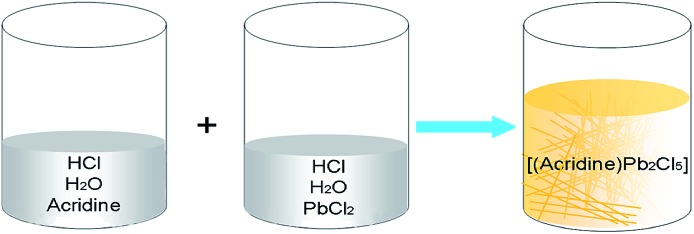
The facile synthesis process of OIHP-AD perovskite crystals in aqueous solution at room temperature.

In this work, it is demonstrated that a one-dimensional (1D) single-crystalline perovskite micro-belt can be immediately isolated using a mixture of AD and lead chloride in an acid aqueous solution (Scheme 1). This is achieved without any organic solvent or expensive alkyl halide. The obtained organic–inorganic hybrid perovskite material [(AD)Pb_2_Cl_5_] (OIHP-AD) can retain its emission for months in an aqueous suspension, thus overcoming the typical instability of hybrid perovskite materials in water. Moreover, the OIHP-AD micro-belt, which possesses high optical gain, can serve as an efficient 1D waveguide material with a low waveguide loss coefficient. Therefore, this work demonstrates a facile, fast, and easily scalable synthesis for 1D organic–inorganic hybrid perovskite photonic materials with high water stability, which may have potential as micro-devices for optical and photonic applications.

## Results and discussion

**Fig. 1:**
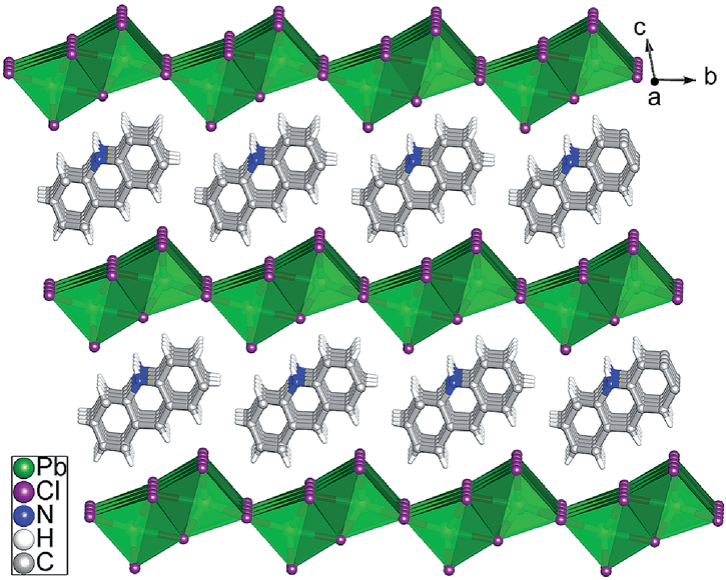
Ball-and-stick/polyhedron view of the OIHP-AD perovskite crystal structure with an alternate arrangement of the inorganic layers and the organic AD layers.

Single crystals suitable for X-ray analysis (Fig. S1 in the ESI†) were obtained using a mixture of AD and PbCl_2_ (1 : 2 molar ratio) in water with addition of hydrochloric acid under hydrothermal conditions and heated at 120 °C for 12 h. Single-crystal X-ray diffraction analysis revealed that [(AD)Pb_2_Cl_5_] crystallizes in a triclinic system with the space group *P*1[combining macron] (Table S1†), which is slightly different from those of typically reported organic–inorganic hybrid perovskite structures.^4^ The asymmetric unit of OIHP-AD consists of one crystallographically independent Pb ion, two half chlorine ions and half a protonated AD cation. As shown in Fig. 1, the crystal structure of OIHP-AD exhibits an alternating arrangement of the organic AD cation layers and the inorganic anion layers constructed by corner-sharing (PbCl_6_) octahedra. Each organic AD layer derived from the (001) crystallographic plane is fixed between adjacent inorganic layers through electrostatic, C–H···Cl and N–H···Cl hydrogen bonds. In addition, π···π stacking between the parallel phenyl and pyridyl rings of AD in an offset fashion with centroid–centroid separations of 3.674 Å can be observed. In such an organic–inorganic hybrid system, the rigid lead chloride inorganic layer can afford a confined and ordered environment, minimizing the aggregation-caused quenching and increasing the optical/thermal stability of AD chromophores. Alternatively, the micro-belt structure of the OIHP-AD (Fig. S2†) can be obtained by a facile precipitation process in an aqueous solution: solutions of AD and PbCl_2_ were separately dissolved with hydrochloric acid and mixed together with stirring. As a result, it could be easily scaled up to the gram level for the 1D OIHP-AD nanocrystals (see the Experimental section, ESI†). Powder X-ray diffraction (PXRD) patterns for the samples isolated under hydrothermal conditions and room temperature correspond well with the simulated data, which illustrates the single phase and high purity of OIHP-AD (Fig. S3 and S4†). The difference in the intensity ratio between some PXRD peaks can be attributed to the preferred orientation of the samples during the measurement. Additionally, the PXRD peaks for the sample obtained under hydrothermal conditions present narrower and stronger peaks than those from room temperature, suggesting a relatively low crystalline degree for the latter.

**Fig. 2:**
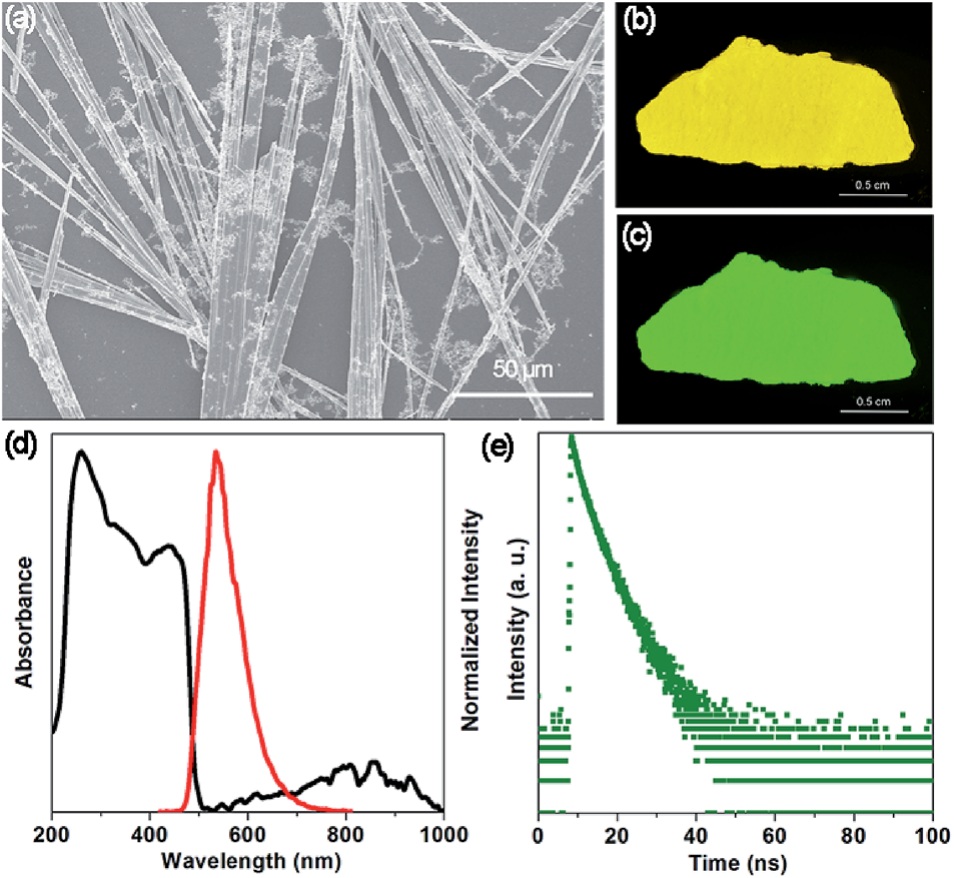
(a) SEM image of the 1D OIHP-AD micro-belts. Photographs of the self-supporting OIHP-AD film (about 0.8 × 3 cm^2^) under (b) daylight and (c) UV (365 nm). (d) UV-vis-NIR absorption (black) and fluorescence emission spectra (red, excitation at 400 nm) of OIHP-AD in the solid state at room temperature. (e) Time-resolved fluorescence decay of OIHP-AD in the solid state.

The SEM image (Fig. 2a) shows that the OIHP-AD sample at room temperature displays a stronger tendency to assemble into a 1D belt-like structure with the length and width in the ranges of 150–500 μm and 1–4 μm, respectively. Due to the high length/diameter aspect ratio of these 1D micro-belts (Fig. S5†), a bright yellow self-supporting OIHP-AD film of several centimeters can be easily achieved (Fig. 2b). Under UV irradiation (*λ*_ex_ = 365 nm), the OIHP-AD film exhibits a bright green emission arising from the highly orientated 1D micro-belt ensemble (Fig. 2c and S6†). To characterize the optical properties of the OIHP-AD films, their UV-vis-NIR absorption, photoluminescence (PL) spectra, PL quantum yield (PLQY) and lifetime were further measured. Fig. 2d presents the UV-vis-NIR absorption and emission spectra of the OIHP-AD film, which displays strong absorption ranging from 200 to 500 nm and a broad absorption peak at 857 nm in the NIR region. However, the pristine AD only exhibits sharp absorption ranging from 200 to 450 nm (Fig. S7a†).

Under ambient conditions, the solid-state luminescence spectrum of AD presents weak blue fluorescence emission at 451 nm (*λ*_ex_ = 330 nm, Fig. S7a†) with a PLQY of 1.43%. This can be ascribed to a strong fluorescence quenching effect between the AD molecules. In contrast, the hybrid 1D OIHP-AD exhibits longer emission wavelength (maxima at 533 nm, *λ*_ex_ = 400 nm) and higher PLQY (7.45%) compared with the pristine AD. The distinct color change from blue to green (red-shift of 82 nm) can be assigned to π···π stacking interactions between adjacent AD moieties together with C–H···Cl and N–H···Cl hydrogen bonds between heterogeneous layers. Moreover, time-resolved fluorescence decay gives a fluorescence lifetime of 2.87 ns for AD and 3.81 ns for OIHP-AD (Fig. 2e and S7b†). The prolonged fluorescence of the OIHP-AD hybrid can be related to the fact that the AD dye is highly restricted and stabilized within the negatively charged inorganic matrix. Benefitting from the confinement effect of the inorganic layers, the molecular thermal vibration and nonradiative relaxation process can be effectively suppressed. The serious aggregation-caused quenching of the chromophores can also be minimized due to the isolation of the OIHP layers.

**Fig. 3:**
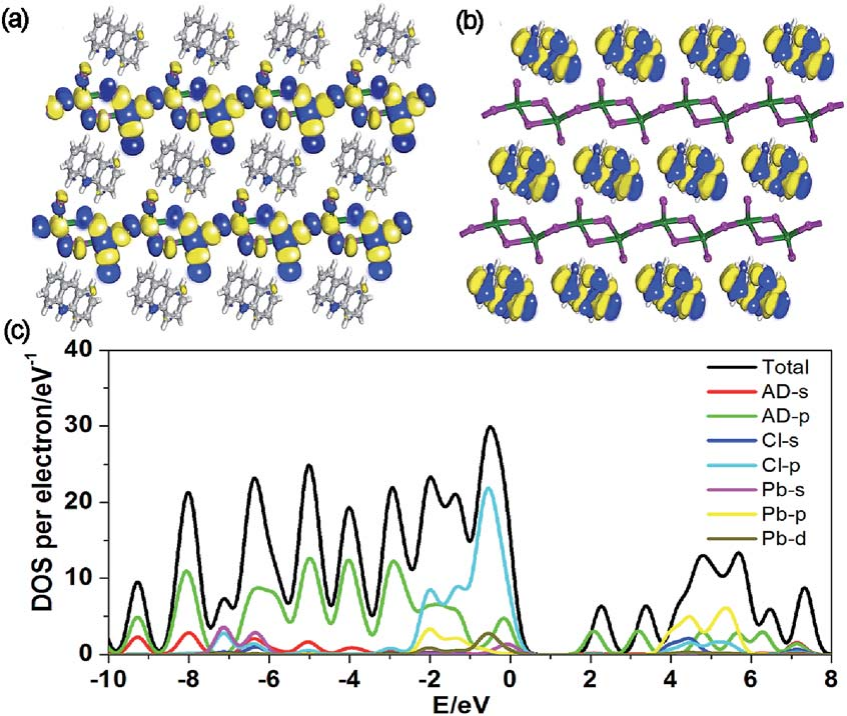
Molecular orbitals of the HOMO (a) and LUMO (b) for OIHP-AD. (c) Total/partial electronic density of states (TDOS and PDOS) for the DFT optimized structure of OIHP-AD.

To better understand the optical properties of OIHP-AD, the band structure, density of states (DOS) and electron-density distribution were determined using periodic density functional theory (DFT) calculations.^12^ The computation shows that OIHP-AD (Fig. S8†) exhibits a low band gap of 2.06 eV, which may correspond to the fluorescence emission around 533 nm (2.33 eV). Frontier orbital analysis further shows that the electron densities of the highest occupied molecular orbital (HOMO) are predominantly localized on the lead chloride inorganic layer, while the electron-density distributions of the lowest unoccupied molecular orbital (LUMO) are observed on the organic AD cation layers (Fig. 3a and b), suggesting potential energy/electron transfer during the photoexcitation process.

Further total electronic density of states (TDOS) and partial electronic density of states (PDOS) analyses (Fig. 3c) reveal that the valence bands (VB) are mainly contributed by the 3p orbitals of Cl and 2p orbitals of C atoms in AD, whereas the conduction bands (CB) are mainly derived from the 6p orbitals of Pb. Therefore, the photoexcitation and emission of OIHP-AD system can be obtained from the lead chloride inorganic layer to the AD organic layers during fluorescence process. Such an electronic structure and photo-physical properties of OIHP-AD may improve electron–hole separation during the charge transfer process, which could correspond to the enhanced PL lifetime in experiments.

**Fig. 4:**
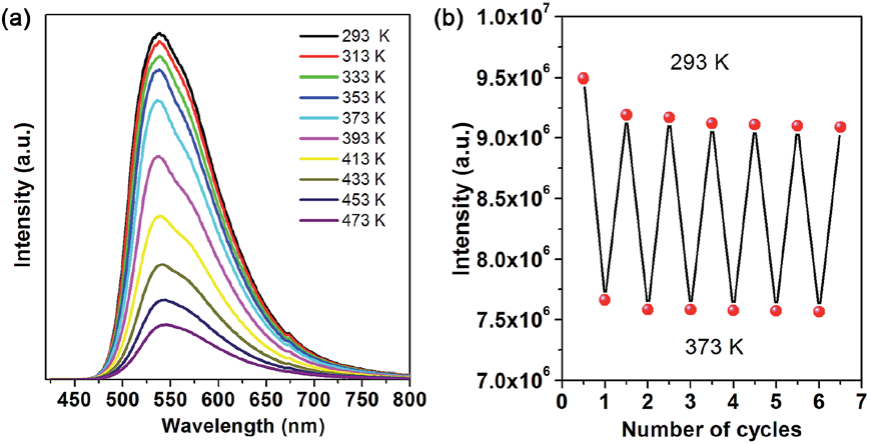
(a) PL spectra of OIHP-AD measured at temperatures from 293 to 473 K. (b) Reversible variation of the PL intensity of OIHP-AD between 293 and 373 K.

As is typical for hybrid perovskite materials, physicochemical stability is a critical issue that restricts their practical application. Among various factors, temperature, moisture and water are considered as major challenges. Thermal gravimetric analysis (TGA) reveals that the as-prepared OIHP-AD remains stable upon heating to higher temperatures (*ca.* 230 °C) compared to the pure AD (111 °C). The weight loss of 25.17% observed from 190 to 300 °C is very close to the calculated value of 23.34%, which corresponds to the AD cation component. With further heating, the remaining substance decomposes quickly until heating to 800 °C (Fig. S9†). Fig. 4a presents the temperature-dependent PL spectra of OIHP-AD. It was observed that the intensity at 533 nm exhibits a slightly decreasing trend from ambient conditions (293 K) to 373 K and decreases obviously upon further increase in temperature from 373 to 473 K. It is worth noting that the PL intensity of OIHP-AD can recover its original position (Fig. 4b) after returning to the initial temperature, confirming the stability of the temperature-dependent luminescence. Moreover, the reversible change in PL can be repeated at least 6 times during heating–cooling cycles between 293 and 373 K, indicating that the 1D OIHP-AD can potentially serve as a luminescent switch for sensing the temperature.

**Fig. 5:**
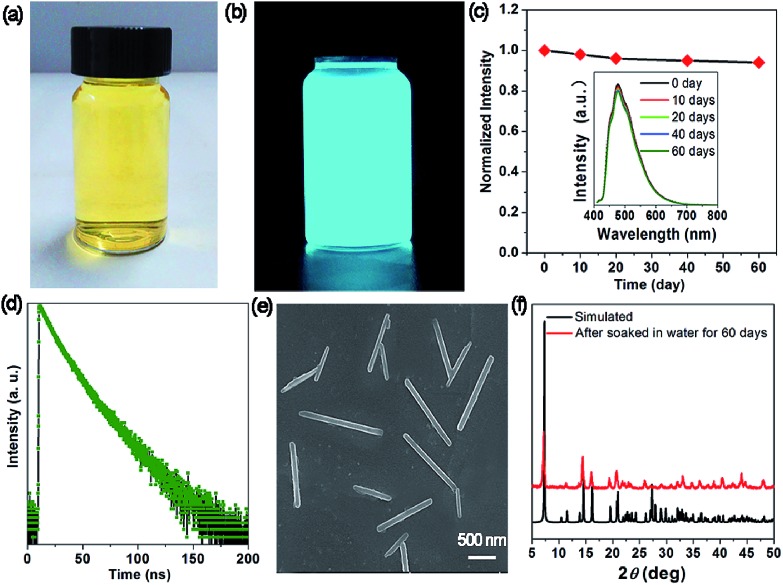
Photographs of the as-obtained OIHP-AD nanocrystal aqueous suspension under (a) ambient conditions and (b) UV light (365 nm) irradiation. (c) Stability of OIHP-AD dispersed in water. The inset shows PL spectra measured at different times (excitation at 409 nm). (d) Time-resolved fluorescence decay of OIHP-AD dispersed in water measured at room temperature. SEM image (e) and PXRD patterns (f) of OIHP-AD after being soaked in water for 60 days.

The measurement of the moisture stability shows that the OIHP-AD self-supporting film can remain unchanged after exposure to a steam atmosphere (90% relative humidity) for 24 h (Fig. S10†). To check the water stability of OIHP-AD, 100 mg of powdered OIHP-AD was added to 20 mL deionized water at room temperature, giving rise to a yellowish suspension (Fig. 5a), which emits a strong cyan light under UV (365 nm) irradiation (Fig. 5b). As shown in Fig. 5c, the fluorescence emission of the OIHP-AD suspension is mainly located at 477 nm (*λ*_ex_ = 400 nm), which is distinctly blue-shifted compared with that of its solid state. It is worth noting that after a long aging time (60 days), there is no obvious decrease in the fluorescence intensity. The time-resolved PL measurement shows that the PL lifetime of the OIHP-AD suspension is 15.03 ns (Fig. 5d); further, the PL quantum yield (PLQY) is 58.79%. After being centrifuged from the suspension, 1D nano-scaled wires can be observed (SEM, Fig. 5e) with the length of 0.5–1.5 μm and width of 100 nm. The PXRD pattern of these nanowires matches well with the simulated pattern of OIHP-AD, indicating that the crystal structure of OIHP-AD nanowires remains unchanged even after soaking in water for more than 60 days (Fig. 5f). It is believed that the blue shift and enhanced PLQY of the 1D nano-scale wires relative to the OIHP-AD self-supporting film can be assigned to the deaggregation of the OIHP-AD along with the decrease in the size of the nanocrystals, as the luminescence of the hybrid nanoparticles is highly dependent on the size, shape, and morphology.7*a* Additionally, when the 1D microbelts were soaked in other typical solvents (such as ethanol, acetone, acetonitrile and chloroform), there is nearly no decrease in the length and width of the 1D microbelts.

To date, although numerous efforts have been made to achieve water-stable perovskite micro- and nano-particles with low dimension (0D, 1D and 2D),4a,^7^,^9^,^13^ most are focused on the external encapsulation of metal oxides,^14^,^15^ polymers,^16^,^17^ mesoporous inorganic oxides^18^,^19^ and metal–organic framework^20^ matrixes. Only recently, Han's and Yang's groups demonstrated that pyridine additives with various alkyl-chain substituents or alkyl ammonium cations on the perovskite surface can substantially improve the stability of perovskite solar cells.^21^,^22^ Different from these surface modifications of the perovskite mentioned above, in this work, the protonated AD cations can act as counter ions evenly distributed between inorganic laminates. Due to their steric hindrance, the coordination capability of AD decreases and thus restricts the reaction with lead ions. Meanwhile, the AD cations are joined together by strong electrostatic, hydrogen bond and π···π stacking interactions, affording denser crystal packing and effective organic water-resisting layers to prevent corrosion due to water molecules to maintain their long-term stability under humid conditions and in water. Therefore, this work may provide a new strategy for the facile synthesis of long-term stable organic–inorganic hybrid perovskite materials by introducing organic cations with larger steric hindrance.

**Fig. 6:**
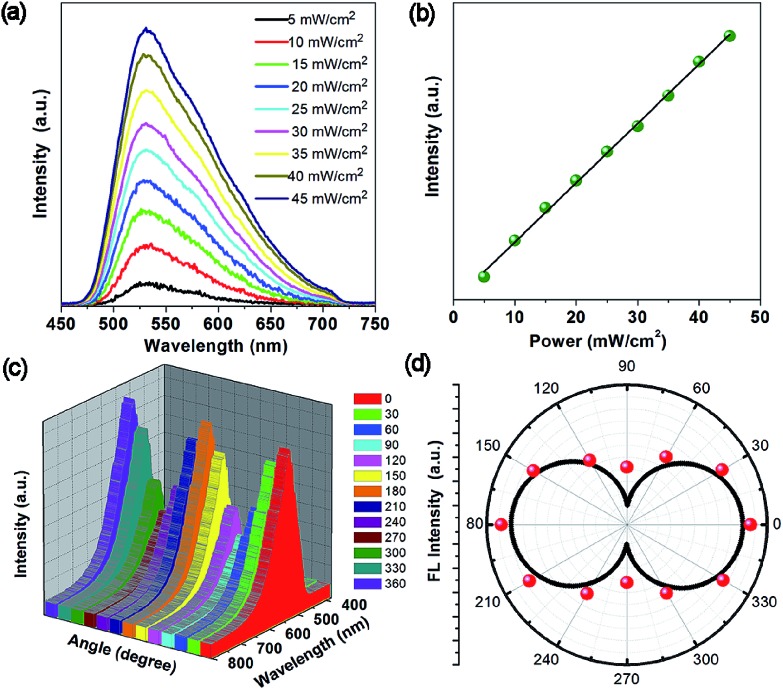
(a) Up-conversion fluorescence spectra of OIHP-AD excited using an 800 nm laser under different pump powers. (b) The changes in intensity with increasing pump powers. (c) Polarized fluorescence spectrum of OIHP-AD at various angles (0–360°). (d) Fluorescence intensity of the OIHP-AD as a function of the polarizer rotation angle.

Due to the high physicochemical stability and excellent optical properties of the 1D OIHP-AD micro/nanocrystals, the upconversion, polarized photoemission and optical waveguide performances are highly desirable for new photonic applications. Encouraged by the broad absorption of OIHP-AD in the NIR region, long-wavelength-excited fluorescence emissions were thus further measured. Upon excitation with a femtosecond pulsed laser at 800 nm, the OIHP-AD exhibits short wavelength fluorescence with an emission peak close to the emission bands obtained by excitation with 400 nm light (Fig. 6a). It can be observed that OIHP-AD displays a nearly linear relationship between the PL intensity and the pump power (Fig. 6b), indicating that the optical gain can be effectively improved by the incorporation of dye cations into the organic–inorganic hybrid perovskite system.

The highly ordered arrangement of AD cations within the rigid lead chloride inorganic matrix further motivates us to study the polarization properties of the perovskite material. The polarized fluorescence spectrum was recorded at the tip of the single micro-belt by rotating the polarizer at different polarization angles. Fig. 6c and d present the relationship between the polarization photoemission intensity of a single micro-belt and the polarization angle. It can be found that the maximum photoemission intensity values for the 1D OIHP-AD micro-belt appear at the cross angles of about 0° and 180°, whereas the minimum ones occur at 90° and 270°. The emission dichroic ratio *R*_d_ and polarization anisotropy *σ* were measured to be about 2.75 and 0.93, respectively, where *R*_d_ = *I*_max_/*I*_min_ and *σ* = (*R*_d_ – 1)/(*R*_d_ + 1).^23^ The polarization anisotropy value for the OIHP-AD 1D micro-belt is higher than that for most reported 1D micro-/nano-sized systems.^24^ These results confirm that the incorporation of dye cations into the perovskite rigid matrix forms a well-oriented and uniformly ordered structure that could potentially act as a polarized luminescent material.

**Fig. 7:**
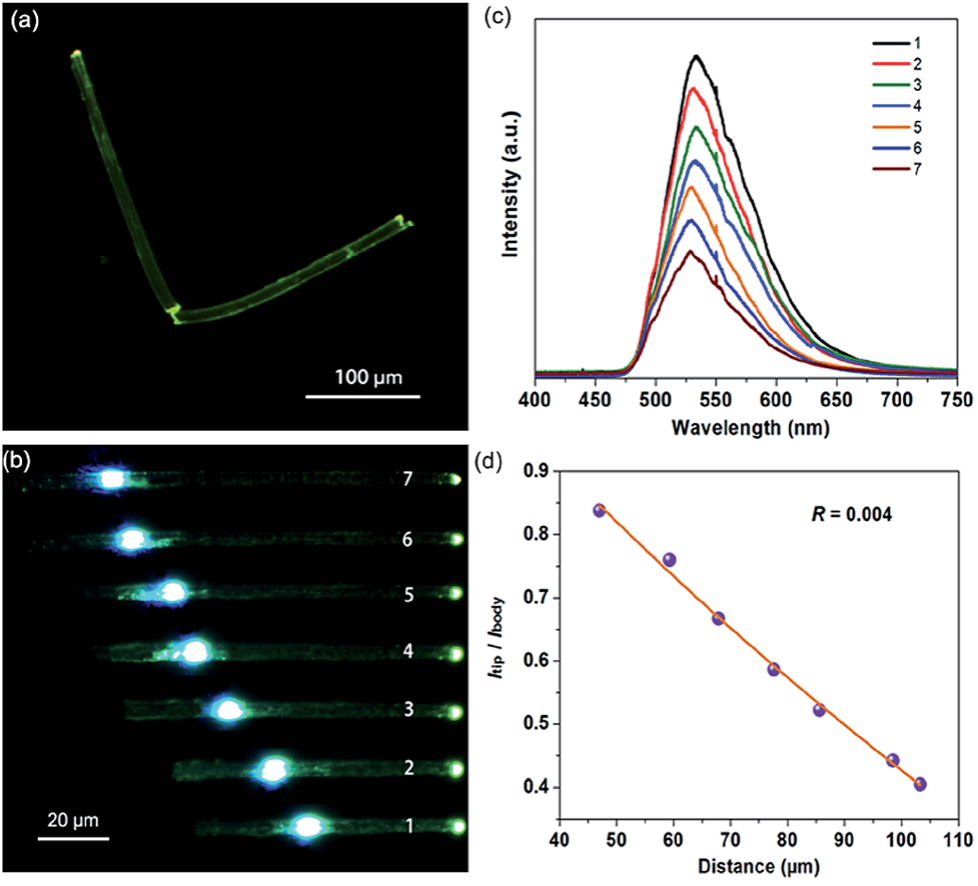
(a) PL microscope images of the OIHP-AD micro-belt under UV (365 nm) light. (b) PL images obtained from a single OIHP-AD micro-belt by exciting at different positions (images 1–7). (c) Spatially resolved PL spectra from the tip of the OIHP-AD micro-belt for different distances between the excitation spot and the tip of the micro-belt shown in PL images 1–7. (d) Optical-loss coefficient (*R*) of the OIHP-AD micro-belt.

When irradiated under unfocused UV light (400 nm) on a PL microscope, the individual OIHP-AD micro-belts show bright green spots on the tips, but relatively weak emission from the belt’s main bodies (Fig. 7a). This observation can be related to the strong optical waveguide effect for the 1D micro-belt, in which the photons transfer from the main body to the ends. This is the intrinsic performance for several 1D/2D luminescent micro/nanostructures: the fluorescence is highly confined inside the crystal and a low-loss optical signal can be obtained during light propagation. Based on such easily observed optical waveguide behavior, further spatially resolved PL imaging (Fig. 7b) and spectroscopy (Fig. 7c) from both the tips and excitation positions were performed by changing the excitation laser beam (400 nm) at different local positions of an individual OIHP-AD micro-belt. During light propagation, the generated photons are confined and propagate along the length of the 1D micro/nanostructure. The emission intensity at the tip decreases almost exponentially with the increase of the propagation distance, while it remains constant at the excited points. To evaluate the waveguide activity of this optical waveguide material, the optical-loss coefficient (*R*) was calculated. Fig. 7d shows the intensity ratio between the tips and bodies (*I*_tip_/*I*_body_) against the propagation distance, which reveals a nearly single-exponential decay. An optical loss coefficient *R* of 0.004 dB μm^–1^ can be obtained by using the function *I*_tip_/*I*_body_ = *A* exp(–*RD*), where *A* is a constant and *D* is the distance between the excited site and the emitting tip.^25^ The excellent optical waveguide properties of this perovskite micro-belt are comparable with those of reported inorganic nanowire and nanofiber waveguides.^26^ This reveals that the optical waveguide efficiency is related to the morphology, crystallinity and luminescent properties of the waveguide.24d,^27^ Here, the relatively low PLQY of the OIHP-AD micro-belt has extremely low optical loss. This phenomenon is similar to the case of the donor–acceptor molecular crystal optical waveguide material.^28^ Conversely, the high optical waveguide performance of OIHP-AD results from the spatial confinement effect of the layer-by-layer nanostructure, which may afford an opportunity to achieve high-quality resonant cavities for micro-lasers.

## Conclusions

In summary, a new 1D single-crystalline micro-belt perovskite material can be fast fabricated by the supramolecular self-assembly of AD cations and lead chloride anionic layers in aqueous solution. The highly regular arrangement of protonated AD in the host–guest system confers enhanced fluorescence performance (such as higher PLQY and lifetime compared with the pristine AD) since the chromophore could be tightly fixed and locked in the rigid framework through non-covalent interactions, thus restricting the vibrations and nonradiative loss. The solid-state perovskite and self-supporting film present high water stability, which can be related to the electrostatic and hydrogen bonding interactions between the host–guest components and the larger steric hindrance of the AD cations distributed between the rigid lead chloride inorganic layers. This can vastly inhibit the ingression of water molecules into the perovskite. Spatially resolved photoluminescence spectra confirm that OIHP-AD micro-belts exhibit a low waveguide loss coefficient (0.004 dB μm^–1^). Therefore, this study not only provides a facile way to develop a new type of low-dimensional organic–inorganic hybrid perovskite material with high water stability but also enables their potential applications in upconversion, polarized photoemission and optical waveguides for the construction of future optical communication micro-devices.

## Conflicts of interest

There are no conflicts to declare.

## Supplementary Material

Supplementary information

Crystal structure data
