# Supplementary material for: Facile synthesis of 1D organic–inorganic perovskite micro-belts with high water stability for sensing and photonic applications
Source: Chem Sci. 2019 Mar 8;10(17):4567–72. doi: 10.1039/c9sc00162j (PMC6492630; doi:10.1039/c9sc00162j)
Supplement: Supplementary file 1 [file SC-010-C9SC00162J-s001.pdf]

Electronic Supplementary Material (ESI) for Chemical Science.
This journal is © The Royal Society of Chemistry 2019

Electronic Supplementary Information

Facile Synthesis of 1D Organic–Inorganic Perovskite Micro-Belts with High Water Stability for Sensor and Photonic Applications

Xiaogang Yang,[ab] Lu-Fang Ma,[b] and Dongpeng Yan*[a]

[a] Beijing Key Laboratory of Energy Conversion and Storage Materials, College of Chemistry, Beijing Normal University, Beijing 100875, P. R. China. E-mail: yandp@bnu.edu.cn

[b] College of Chemistry and Chemical Engineering, Henan Province Function-oriented Porous Materials Key Laboratory, Luoyang Normal University, Luoyang 471934, P. R. China.

A. Experimental Section

1. Materials and general procedures.

Analytically pure $PbCl_2$, and acridine (AD) were commercial available, and used without further purification. Single-crystal X-ray diffraction data were collected at room temperature on an Oxford Diffraction SuperNova area-detector diffractometer using mirror optics monochromated Mo Kα radiation (λ = 0.71073 Å). CrysAlisPro[1] was used for the data collection, data reduction and empirical absorption correction. The crystal structure was solved by direct methods, using SHELXS-2014 and least-squares refined with SHELXL-2014[2] using anisotropic thermal displacement parameters for all non-hydrogen atoms. The crystallographic data for OIHP-AD are listed in Table S1. CCDC No. 1862717 contain the supplementary crystallographic data for OIHP-AD. The data can be obtained free of charge from The Cambridge Crystallographic Data Centre via http://www.ccdc.cam.ac.uk/data_request/cif. Other data supporting the findings of this study are available from the corresponding author. Powder X-ray diffraction analyses (PXRD) patterns were collected on a Rigaku Ultima-IV automated diffraction system with Cu Kα radiation (λ = 1.5406 Å). Measurements were made in a 2θ range of 5−50° at room temperature with a step of 0.02° (2θ) and a counting time of 0.2 s/ step. The operating power was 40 KV, 30 mA. Thermogravimetric analysis (TGA) experiments were carried out using a Perkin-Elmer Diamond SII thermal analyzer from room temperature to 800 ^{o}C under a nitrogen atmosphere at a heating rate of 10 ^{o}C min^{-1}.

Photographs of the OIHP-AD self-supporting film under UV excitation (365 nm) and ambient conditions were captured using a Canon digital camera (EOS 700D: the ISO value was 400, time of exposure was set to an automatic mode). PL microscope images of OIHP-AD micro-belts were taken under OLYMPUS IXTI fluorescence microscope. The morphology of the samples were investigated by using a scanning electron microscope (SEM Hitachi S-3500) equipped with an energy dispersive X-ray spectrum attachment (EDX Oxford Instruments Isis 300), with an acceleration voltage of 20 kV. Room temperature photoluminescence (PL) spectra and time-resolved lifetime were conducted on an Edinburgh FLS980 fluorescence spectrometer

equipped with a xenon arc lamp (Xe900) and nanosecond flash-lamp (nF900), respectively. The PLQY values at room temperature were estimated using an integrating sphere (F-M101, Edinburgh) accessory in FLS980 fluorescence spectrometer. The temperature dependence of the PL spectra were measured using a temperature controller attached to a cryostat (Oxford Ltd. Optistat DN2) using an FLS980 fluorescence spectrometer. Optical diffuse reflectance spectra were obtained on a Shimadzu UV-3600 spectrophotometer at room temperature. Data were collected in the wavelength range of 200−800 nm. $BaSO_4$ powder was used as a standard (100% reflectance). Upconversion fluorescence PL spectrum was excited by 800 nm laser on a Tsunami-Spitfire-OPA-800C ultrafast optical parameter amplifier (Spectra Physics). Polarized fluorescence spectrum of the single micro-belt were measured on a CRAIC 508PV microspectrometers (λ_{ex} = 420 nm) by rotating the polarizer at different polarization angle. Spatially resolved PL imaging and spectroscopy of the single micro-belt were taken from a CRAIC 508PV microspectrometers excited by a focused 400 nm laser beam, which was propagated by a optical fiber.

2. Preparations of OIHP-AD samples.

2.1 Preparations of OIHP-AD single crystal.

In a typical synthesis, mixture of AD (0.25 mmol), $PbCl_2$ (0.5 mmol) and HCl (1 M, 1 mL) were added to water (12 mL) in a 25 mL Teflon-lined stainless steel vessel, and then heated at 120 ^{o}C for 12 h. After the reactive mixture was slowly cooled to room temperature, bright yellow belt-like crystals of OIHP-AD were obtained.

2.2 Preparations of OIHP-AD micro-belt.

Addition of a solution of $PbCl_2$ (0.5 mmol) and HCl (1 M, 0.5 mL) in H_2O (5 mL) to a solution of AD (0.25 mmol) and HCl (1 M, 0.5 mL) in H_2O (5 mL) with vigorous string. Then, bright yellow micro-belt crystals of **OIHP-AD** were obtained immediately.

2.3 Gram-scale synthesis of OIHP-AD micro-belt.

Addition of a solution of $PbCl_2$ (5 mmol) and HCl (1 M, 5 mL) in H_2O (20 mL) to a solution of AD (2.5 mmol) and HCl (1 M, 5 mL) in H_2O (20 mL) with vigorous string in a vial (50 mL). Then, bright yellow micro-belt crystals of OIHP-AD were obtained immediately.

3. Electronic structure calculations of OIHP-AD

All calculations were performed with the periodic density functional theory (DFT) method using Dmol3 [3] module in Material Studio software package.[4] The initial configuration was fully optimized by Perdew-Wang (PW91) [5] generalized gradient approximation (GGA) method with the double numerical basis sets plus polarization function (DNP). The core electrons for metals were treated by effective core potentials (ECP). The self-consistent field (SCF) converged criterion was within 1.0×10^{-5} hartree atom^{-1} and the converging criterion of the structure optimization was 1.0×10^{-3} hartree bohr^{-1}. The Brillouin zone is sampled by $1 \times 1 \times 1$ *k*-points, and test calculations reveal that the increase of *k*-points does not affect the results.

B. Supporting Figures

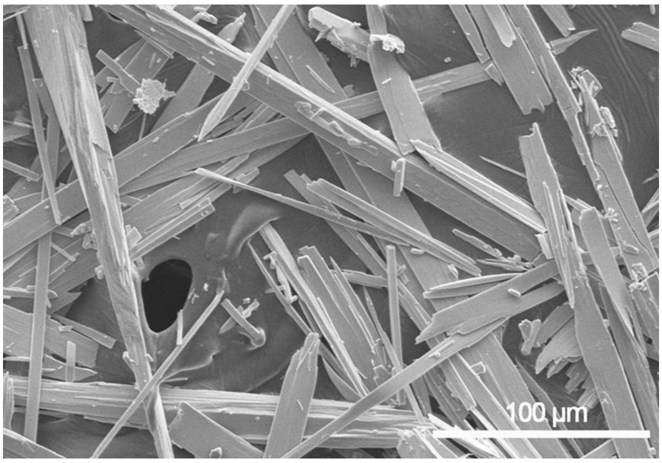

Figure S1. SEM image of the as-prepared OIHP-AD crystals synthesized under hydrothermal condition.

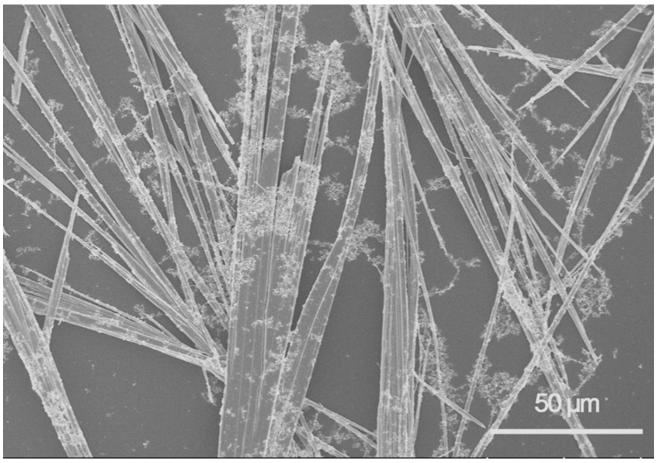

Figure S2. SEM image of the as-prepared OIHP-AD crystals synthesized in aqueous solution at room temperature.

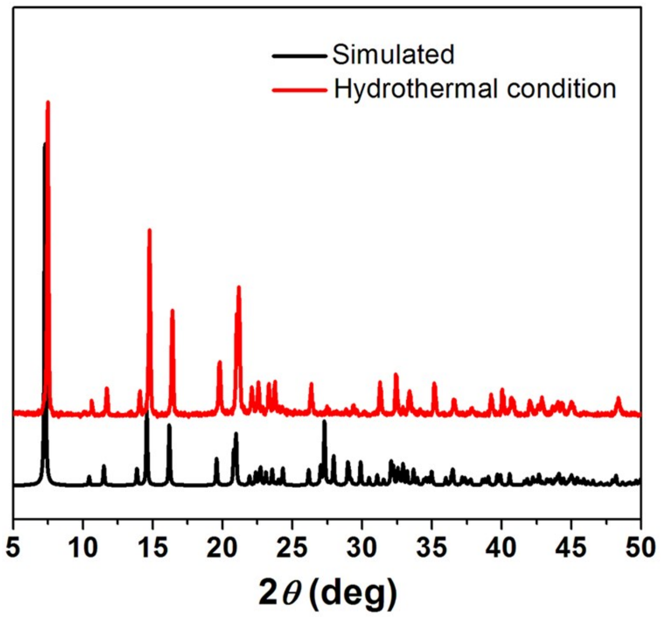

Figure S3. PXRD patterns of simulated (black) and experiment OIHP-AD (red) synthesized under hydrothermal condition.

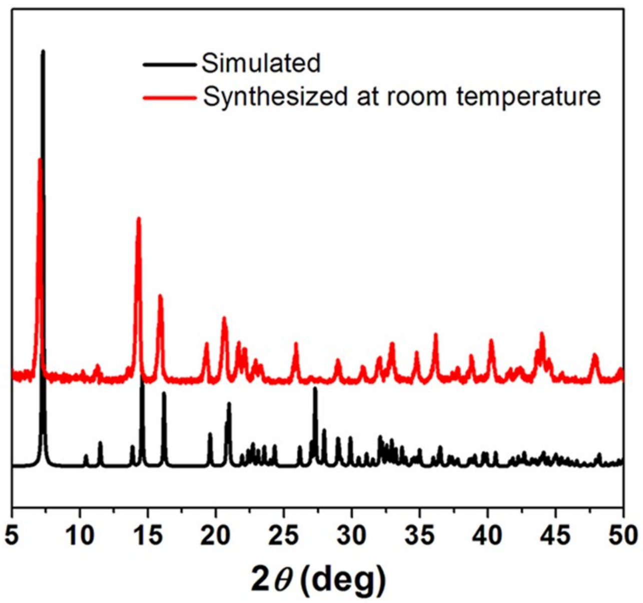

Figure S4. PXRD patterns of simulated (black) and experiment OIHP-AD (red) synthesized in aqueous solution at room temperature.

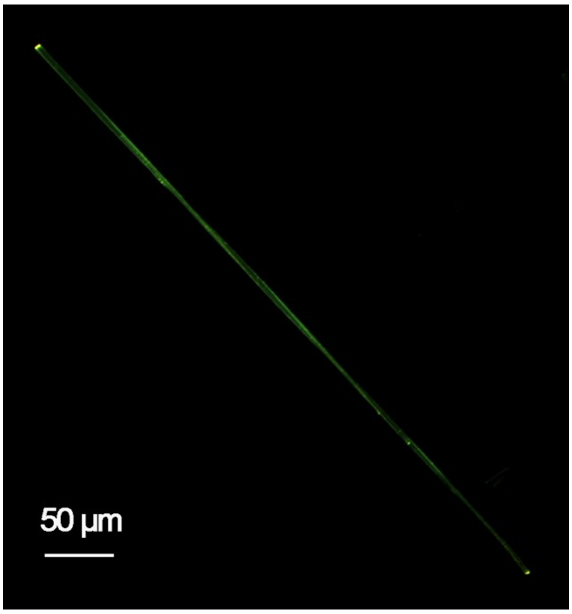

Figure S5. Photoluminescence microscope image of single OIHP-AD micro-belt.

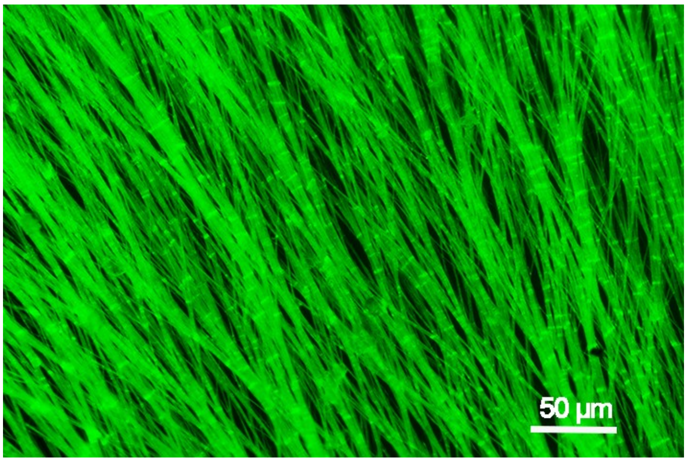

Figure S6. Photoluminescence microscope images of OIHP-AD self-supporting film.

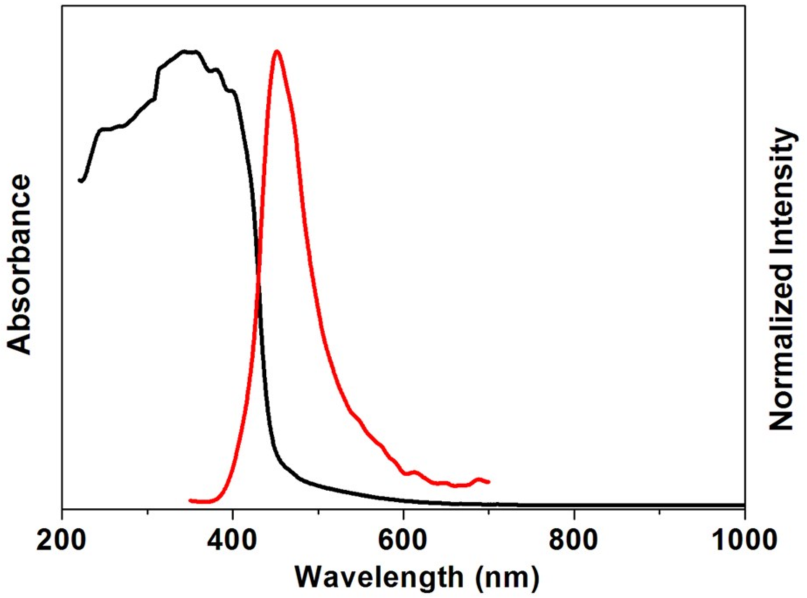

(a)

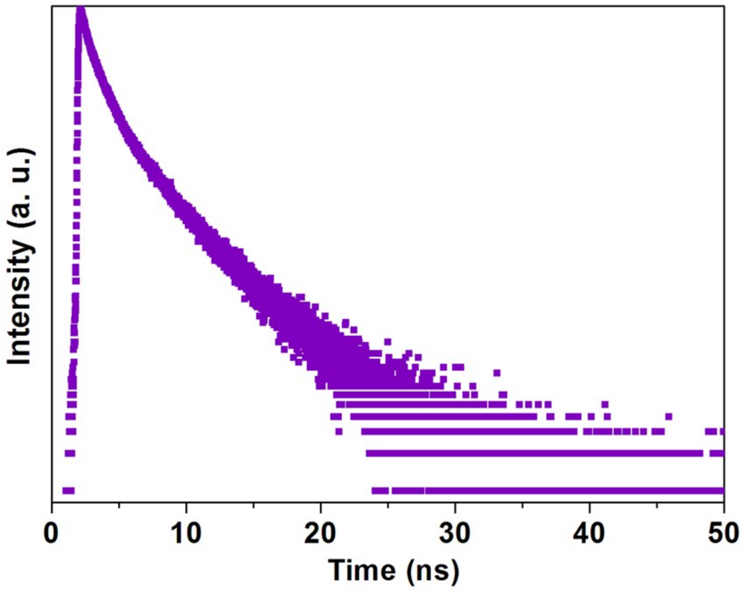

(b)

Figure S7. (a) UV-vis absorption (black) and fluorescence emission spectrum (red, excitation at 280 nm) of AD in solid state at room temperature. (b) Time-resolved fluorescence decay of AD in solid.

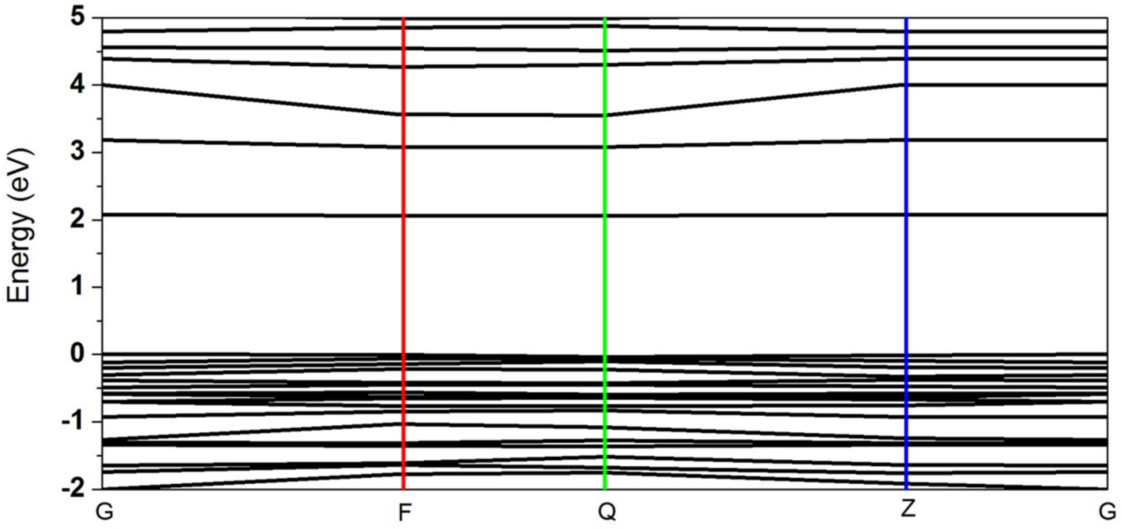

Figure S8. Calculated energy band structure of OIHP-AD.

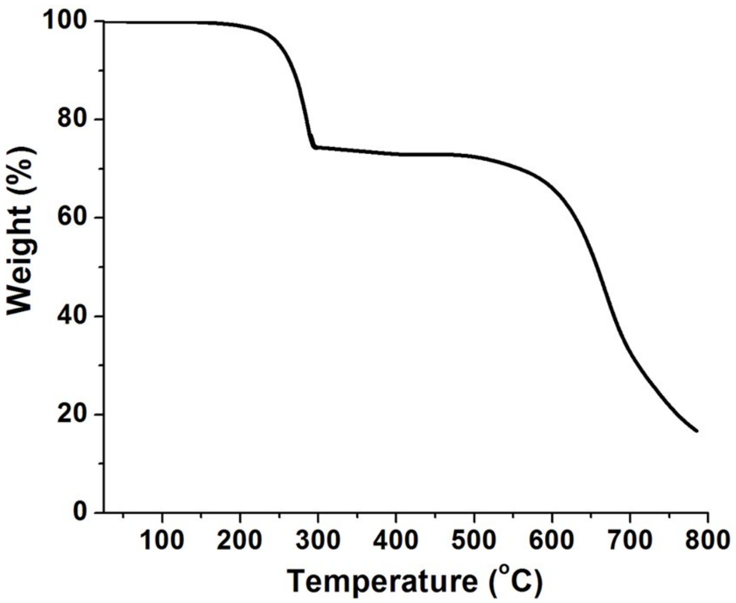

Figure S9. Thermogravimetric analysis (TGA) curves of OIHP-AD crystals.

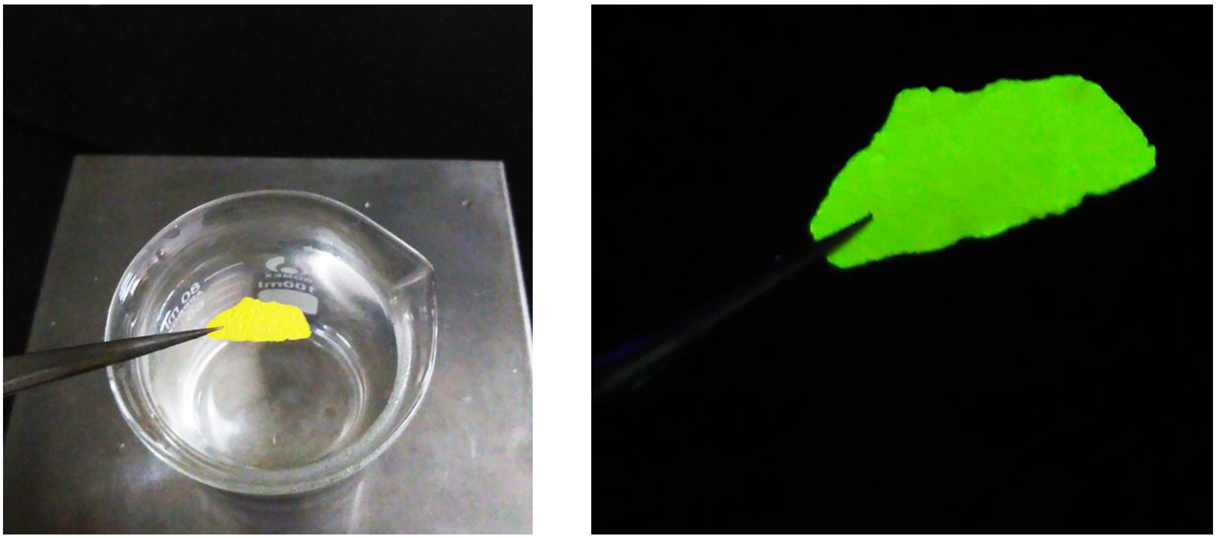

(a) (b)

Figure S10. (a) Photographs of OIHP-AD self-supporting film exposed to steam for 24 h and (b) further radiated under UV (365 nm) light.

C. Supporting Tables

Table S1. Crystallographic data for OIHP-AD.

**Samples**	OIHP-AD
Chemical formula	$C_{6.5}H_5N_{0.5}Cl_{2.5}Pb$
Formula weight	386.96
Crystal system	Tricilinic
Space group	$P\bar{1}$
a (Å)	4.0291(3)
b (Å)	8.8371(6)
c (Å)	12.3963(10)
α (°)	100.672(6)
β (°)	91.860(6)
γ (°)	102.273(6)
V (Å^3)	422.64(5)
Z	2
D (g cm^{-3})	3.0404
μ (mm^{-1})	20.678
T (K)	296(2)
R_{int}	0.0720
Goof	0.943
R_1 ($I>2\sigma(I)$)	0.0477
wR_2 ($I>2\sigma(I)$)	0.0549

$R_1 = \Sigma||F_o| - |F_c||/\Sigma|F_o|$, $wR_2 = [\Sigma w(F_o^2 - F_c^2)^2/\Sigma w(F_o^2)^2]^{1/2}$

D. Supporting References

[1] CrysAlisPro, Rigaku Oxford Diffraction, Version 1.171.39.6a.

[2] G. M. Sheldrick, *Acta Crystallogr. Sect. A*. 2008, **64**, 112.

[3]. *a*) B. Delley, *J. Chem. Phys*. 1990, **92**, 508; *b*) B. Delley, *J. Chem. Phys*. 2000, **113**, 7756.

[4] Dmol3 Module, MS Modeling, Version 2.2; Accelrys Inc.: San, Diego, 2003.

[5] J. P. Perdew, J. A. Chevary, S. H.Vosko, K. A. Jackson, M. R. Pederson, D. J. Singh, C. Fiolhais, *Phys. Rev. B*. 1992, **46**, 6671.
